# Tracheal intubation in the thyroid surgical position improves recurrent laryngeal nerve monitoring: a dual-center randomized trial

**DOI:** 10.1097/JS9.0000000000003481

**Published:** 2025-09-12

**Authors:** Enci Liu, Junjie Qin, Xiaoyu Li, Yong Qi, Lingzhi Wang, Xianjiang Wu, Zhanbo Yi, Junping Chen, Bo Lu

**Affiliations:** a Department of Anaesthesiology, Ningbo No. 2 Hospital, Ningbo University and Hangzhou Medical College, Zhejiang Province, China; b Department of Anaesthesiology, Ningbo Medical Centre Lihuili Hospital, Ningbo, Zhejiang Province, China; c Department of Thyroid Surgery, Ningbo No. 2 Hospital, Ningbo University and Hangzhou Medical College, Zhejiang Province, China

**Keywords:** electromyography, intraoperative neuromonitoring, recurrent laryngeal nerve, thyroid surgery, tracheal intubation

## Abstract

**Background:**

Intraoperative neuromonitoring (IONM) is a critical adjunct in thyroid surgery to preserve recurrent laryngeal nerve function. This study aims to evaluate whether tracheal intubation in the thyroid surgical position enhances IONM signal quality compared with the standard supine position.

**Methods:**

In this dual-center, parallel-group, randomized trial, 184 adults scheduled for elective thyroidectomy requiring IONM were allocated 1:1 to intubation in either the thyroid surgical position or the conventional supine position. The modified intention-to-treat cohort comprised 167 participants (85 vs 82) with an assessable baseline vagus response (V1). The primary outcome was the incidence of satisfactory electromyography (EMG) signals, defined as an initial vagus nerve EMG amplitude exceeding 500 μV when stimulated at 1.0 mA. Secondary outcomes included EMG signal amplitude, intubation-related metrics (e.g., Cormack–Lehane grades, intubation difficulty scale scores, intubation time, and depth of intubation), postoperative complications (e.g., lip lesions, dental injury, postoperative nausea and vomiting, dizziness, and headache), and clinician satisfaction with patient positioning and mask ventilation.

**Results:**

Satisfactory V1 signals occurred in 82/85 patients (96.47%) in the thyroid surgical position group versus 70/82 (85.37%) in the supine group (relative risk 1.13; 95% CI 1.03–1.24; *P* = 0.0145). Intubation time and Cormack–Lehane grades did not differ significantly, but the thyroid surgical position group required deeper EMG tube insertion (22 cm [21–23] vs 22 cm [21–22]; median differences 1, 95% CI 0–1; *P* = 0.046). Postoperative complications did not differ significantly between groups (all *P* > 0.05). Clinician satisfaction scores were significantly higher in the thyroid surgical position group (median 9 vs 5; median differences 4, 95% CI 3–4; *P* < 0.0001). Mask ventilation satisfaction was also rated higher in the thyroid surgical position group (median 9 vs 8; median differences 1, 95% CI 0–1 *P* < 0.0001).

**Conclusions:**

Tracheal intubation in the thyroid surgical position significantly improves IONM signal quality and clinician satisfaction without increasing intubation difficulty or postoperative complications. Adopting this simple positioning strategy can optimize EMG tube placement and enhance the reliability of IONM during thyroid surgery.

**HIGHLIGHTS**

- Improved IONM signal quality with thyroid surgical position intubation.
- Equivalent intubation feasibility compared to the conventional supine position.
- Higher clinician satisfaction in airway management and procedural workflow.

## Introduction

Thyroid cancer represents a major global health challenge, with age-standardized incidence rates increasing from 14.06 per 100 000 in 1990 to 22.02 per 100 000 in 2019 globally, corresponding to an estimated annual percentage change of 1.63%^[1]^. Recurrent laryngeal nerve (RLN) paralysis is a common complication associated with thyroid surgery. Jeannon *et al* indicate that approximately 10% of patients undergoing thyroid surgery may experience temporary RLN injury, with one in 25 patients developing persistent voice problems^[2]^. Protecting the laryngeal nerves during thyroid and parathyroid surgeries remains a critical and challenging objective.

The utilization of intraoperative neuromonitoring (IONM) technology as an adjunctive tool has positively contributed to the preservation of nerve function^[3]^. Over the past three decades, IONM has been increasingly adopted worldwide as a standardized method. Accurate positioning of the electromyography (EMG) tracheal tube is essential for successful IONM^[4,5]^. Improper placement of the EMG tracheal tube can result in false negatives or diminished EMG signals^[4]^, adversely affecting the accuracy of surgical decisions. Existing studies have shown that the position of the tracheal tube can vary with changes in patient positioning (such as neck extension, flexion, and rotation)^[6,7]^. However, it remains unclear whether neck extension induced by the thyroid surgical position leads to the displacement of the EMG tracheal tube and whether such displacement impacts the quality of IONM signals or increases the likelihood of needing to reposition the electrodes.

We hypothesize that performing tracheal intubation in the thyroid surgical position enhances IONM signal quality by optimizing the positioning of the EMG tracheal tube relative to the vocal folds. The primary objective of this study is to assess the incidence of satisfactory EMG signals during tracheal intubation in the thyroid surgical position. The secondary objective is to evaluate the amplitude of EMG signals and the feasibility of completing tracheal intubation in this position, including its impact on mask ventilation and clinician satisfaction.

## Materials and methods

### Ethical approval and study registration

We conducted an investigator-initiated, assessor-blinded, dual-center, randomized controlled trial at two tertiary hospitals in Ningbo, China. The study protocol was approved by the Institutional Review Board of Ningbo No. 2 Hospital (Approval No.: PJ-NBEY-KY-2024-131-02) and Ningbo Medical Centre Lihuili Hospital (Approval No.: KY2024PJ459). The trial has been registered in the Chinese Clinical Trial Registry (Registration No.: ChiCTR2400091542). The study adhered to the Consolidated Standards of Reporting Trials (CONSORT) guidelines and followed the principles of the 2013 Declaration of Helsinki and its later amendments. Written informed consent was obtained from all participants or their legally authorized representatives prior to enrollment in the study. This study was reported in accordance with the Transparency in the reporting of artificial intelligence – the TITAN guideline^[8]^. No artificial intelligence tools were used in study design, data collection/analysis, or manuscript preparation.

### Study population

A total of 184 patients scheduled for elective thyroid surgery requiring IONM were enrolled between November 2024 and March 2025. We included patients who were over 18 years old, classified as American Society of Anesthesiologists (ASA) physical status I or II, had a BMI between 18 and 28 kg/m^2^, and required IONM during thyroid surgery. Exclusion criteria included anticipated difficult airways (Mallampati class > III, thyromental distance < 6 cm, or limited neck extension), high risk of aspiration (e.g., hiatal hernia, gastroparesis), and known allergies to anesthetic agents used in the protocol.

### Randomization and blinding

Participants were randomized in a 1:1 ratio to either the supine position group or the thyroid surgical position group by means of a computer-generated random sequence prepared by the study statistician. An independent study physician who was not involved in participant recruitment or outcome assessment prepared the envelopes and performed the group allocation after obtaining written informed consent. Allocation codes were inserted into sequentially numbered, opaque, sealed envelopes, which were opened only after written consent had been obtained and immediately before anesthesia induction. Because patient posture is visually evident, the attending anesthetist and surgeon were necessarily unblinded; researchers responsible for data collection and analysis were blinded to group assignments (except intubation-related metrics). Perioperative data were recorded on case-report forms bearing study IDs only, EMG signal-related data were exported under the same IDs, and a single investigator (BL) who was not involved in airway management independently reviewed the deidentified recordings. Statistical analysis was performed on a coded database, and the allocation key was released only after all primary analyses were completed.

### Anesthetic management

All patients fasted for 8 hours and refrained from fluid intake for 4 hours prior to surgery. Upon arrival in the operating room, intravenous access was established via the great saphenous vein, and routine monitoring was initiated, including ECG, blood pressure, SpO_2_, and bispectral index (BIS).

**Figure 1.**
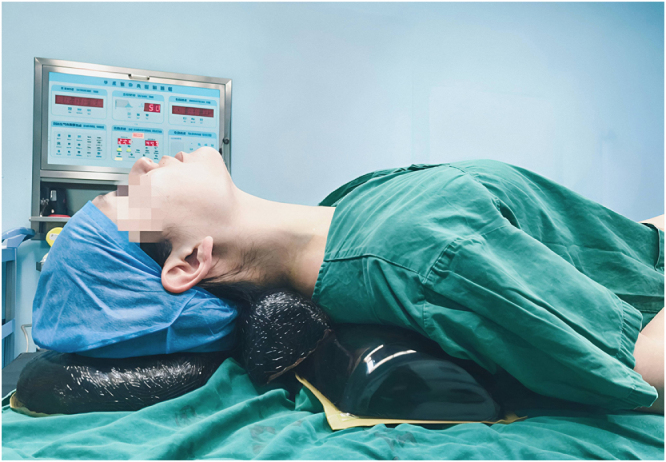
Prior to anesthesia induction, patients were positioned using a specialized surgical pillow to establish the thyroid surgical position. Consent has been obtained from the individual depicted in the image.

During anesthetic induction, patients in the supine position group remained in the standard supine position, while those in the thyroid surgical position group were positioned with a 10 cm soft pillow under the shoulders to elevate the upper back by 45°–55°, a 5-cm neck roll under the neck, and a head ring supporting 30° of neck extension. The chin, trachea, and sternum were aligned on the same horizontal plane (Fig. 1).

Anesthesia was induced with propofol (2–2.5 mg·kg^−1^), sufentanil (0.3 μg·kg^−1^), and rocuronium (0.3 mg·kg^−1^). After 3-minute preoxygenation with 100% O₂ via a face mask, tracheal intubation was performed using a video laryngoscope (Youyi Medical Device Co., Zhejiang, China). An EMG tracheal tube (ID 7.0 mm for males, ID 6.5 mm for females; Baining Yingchuang Medical Technology Co., Jiangsu, China) was placed by an experienced anesthetist.

Anesthesia was maintained with 1–2% sevoflurane and a continuous intravenous infusion of remifentanil (0.1 μg·kg^−1^·min^−1^), to maintain BIS values between 40 and 60. Hypotension (<90 mmHg) was managed with intravenous noradrenaline (4 μg/bolus), and bradycardia (heart rate <50 bpm) was treated with atropine (0.5 mg/bolus).

At the conclusion of the procedure, sevoflurane and remifentanil were discontinued. Postoperative analgesia was provided with sufentanil (10 µg total dose), and ondansetron (4 mg total dose) was administered to prevent nausea and vomiting. Patients were transferred to the Post-Anaesthesia Care Unit for 30 min of observation post-extubation and were transferred to the ward once their modified Aldrete score reached ≥ 9.

### Intraoperative neuromonitoring

Under video laryngoscopic guidance, the midpoint of the surface electrode on the EMG tracheal tube was precisely positioned in direct contact with the vocal folds to record EMG generated by the intrinsic laryngeal muscles. The tracheal tube and its circuit tubing were securely fastened to ensure stability during the procedure. Subcutaneous needle electrodes were placed approximately 1 cm apart in the deltoid region and meticulously secured to minimize movement and ensure consistent signal acquisition.

Once all equipment connections were established, the monitoring system (Model: BN-JCY-100, Youli Biotechnology Co., Ltd, Hunan, China) was powered on, and electrode impedance was assessed. An abnormal impedance (impedance > 5 kΩ) reading indicated poor electrode-vocal fold contact or issues with grounding electrode placement, necessitating immediate readjustment to optimize signal quality. Following impedance verification, the monitoring interface was configured, and the stimulus current and other parameters were adjusted as needed.

After completion of surgical field preparation and exposure of the vagus nerve by opening the carotid sheath, a 1.0 mA current was applied to stimulate the vagus nerve to elicit EMG signals. If the initial vagus nerve EMG amplitude exceeded 500 μV, it was determined that the IONM system was successfully established, with satisfactory signal quality and stable operation, and the amplitude value was recorded simultaneously. If the initial EMG amplitude failed to reach 500 μV, two blinded electrophysiologists immediately conducted a re-evaluation. After consensus confirmation of inadequate amplitude by both experts, this condition was recorded as “unsatisfactory” along with the specific amplitude value. Subsequently, to ensure the quality of subsequent intraoperative monitoring, the tape securing the endotracheal tube was loosened, and a video laryngoscope was used to reposition the monitoring catheter, ensuring full adherence of the vocal cords to the four electrodes on the catheter. A retest was performed after repositioning to confirm the accuracy and reliability of monitoring throughout the entire procedure. Critically, all impedance verification and EMG signal acquisition occurred after patients were positioned in the thyroid surgical posture, regardless of initial intubation position.

### Outcome measures

The primary outcome was the incidence of satisfactory EMG signals, defined as an initial vagus nerve EMG amplitude exceeding 500 µV when stimulated at 1.0 mA. Secondary outcomes encompassed intubation-related metrics, postoperative complications, and clinician satisfaction with patient positioning and mask ventilation.

Intubation-related parameters included the Cormack–Lehane grade (Grade I/II: easy; Grade III: moderate; and Grade IV: difficult, with the epiglottis not visualized), the Intubation Difficulty Scale (IDS) score (0: easy; 0 < IDS ≤ 5: mild difficulty; and IDS > 5: moderate-to-severe difficulty), total intubation time (from laryngoscope insertion to the first end-tidal CO₂ waveform), depth of intubation (measured from the incisors to the tip of the tracheal tube), and the number of intubation attempts. Immediate post-intubation complications, such as oral mucosal bleeding and dental injury, were recorded.

Postoperative complications, including headache, dizziness, nausea, and vomiting, were assessed on postoperative day 1. Clinician satisfaction was evaluated using a 10-point Likert scale, with separate ratings for mask ventilation (by the anesthetist) and patient positioning (by the surgical team), ranging from 1 (“least satisfied”) to 10 (“most satisfied”). Mask ventilation satisfaction specifically measured the anesthetist’s ease and effectiveness of airway management, while patient positioning satisfaction reflected the surgical team’s satisfaction with the process of positioning the patient.

### Statistical analysis

Sample size was calculated with PASS 15 software (NCSS, LLC, Kaysville, UT, USA). With *α* = 0.05 (two-sided) and power (1 − *β*) = 0.8, and assuming a 1:1 allocation ratio, 72 participants were required per group to detect the expected difference in satisfactory EMG signal (80% in the supine group vs 95% in the thyroid position group). Allowing for a 20% dropout rate, the final target sample size was 92 per group (total = 184).

Data analyses were conducted using R 4.1.2 (R Foundation for Statistical Computing, Vienna, Austria) and GraphPad Prism 9.5 (GraphPad Software, Inc., San Diego, CA, USA). All efficacy analyses followed a modified intention-to-treat principle, prospectively defined in the protocol as including every randomized participant for whom an initial vagus-nerve EMG amplitude (V1) was recorded. Continuous variables were assessed for normality via the Shapiro–Wilk test and *Q*–*Q* plots. Parametric data (mean ± SD) were compared using independent *t*-tests; non-parametric data (median [IQR]) with Mann–Whitney *U* tests. Categorical variables (*n* [%]) were analyzed using *χ*^2^ or Fisher’s exact tests. Effect sizes are reported as relative risk (RR) with 95% confidence intervals (CIs) for proportions and or Hodges–Lehmann median differences for non-parametric comparisons. A *post hoc* multivariable Poisson regression was performed to assess potential confounding effects of age, gender, and height on the primary outcome, with results reported as adjusted RR and 95% CI. All tests were two-tailed with *α* = 0.05 defining statistical significance.

## Results

### Patient characteristics

**Figure 2.**
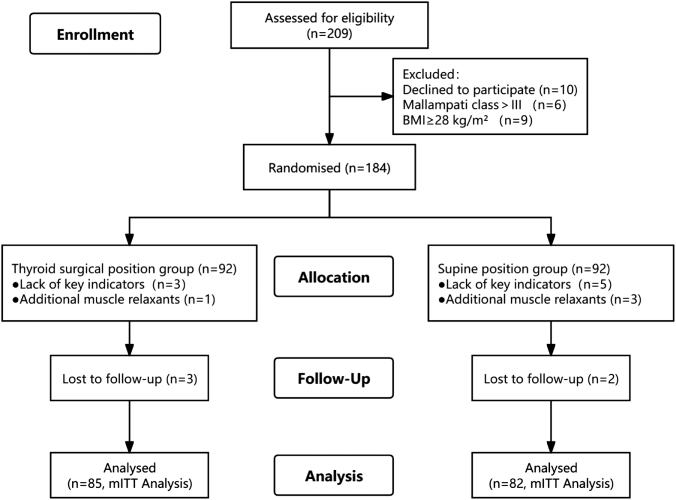
CONSORT flow diagram showing enrollment, allocation, follow-up, and analysis. mITT: modified intention-to-treat.

Among the 209 patients assessed for eligibility, 25 were excluded due to declining to participate (*n* = 10), Mallampati class >III (*n* = 6), or BMI ≥ 28 kg·m^−2^ (*n* = 9), resulting in 184 patients who were randomized into 2 groups: 92 in the thyroid surgical position group and 92 in the supine position group. Owing to the lack of key indicators and the addition of neuromuscular blocking drugs, 85 patients in the thyroid surgical position group and 82 patients in the supine position group were ultimately included in the analysis (Fig. 2).

Baseline characteristics, including age, sex, height, weight, BMI, ASA physical status, surgical side, and Mallampati score, were comparable between groups (all *P* > 0.05; Table 1).

**Table 1:** Baseline demographic, surgical, and anesthetic characteristics

Variable	Thyroid surgical position (*n*=85)	Supine position (*n*=82)	*P*-value
Age, years	44.41 ± 13.46	47.77 ± 14.32	0.1204
Female	63 (74.12%)	64 (78.05%)	0.59
Height, cm	162.90 ± 8.00	162.00 ± 7.05	0.4068
Weight, kg	60.52 ± 10.12	59.72 ± 8.67	0.5855
BMI, kg/m^2^	22.73 ± 2.99	22.71 ± 2.39	0.9572
ASA physical status			0.9923
I	55 (64.71%)	53 (64.63%)	
II	30 (35.29%)	29 (35.37%)	
Surgical side			0.886
Right	40(47.06%)	38(46.34%)	
Left	35(41.18%)	32(39.02%)	
Bilateral	10(11.76%)	12(14.63%)	
EMG acquisition side			0.7038
Left	40 (47.06%)	41 (50.00%)	
Right	45 (52.94%)	41 (50.00%)	
Mallampati class			0.966
I	22 (25.88%)	21 (25.61%)	
II	57 (67.06%)	56 (68.29%)	
III	6 (7.06%)	5 (6.10%)	

Data are presented as mean ± SD or number (%). ASA, American Society of Anesthesiologists; BMI, body mass index.

### IONM and airway management

As shown in Table 2, the incidence of satisfactory EMG signals was significantly higher in the thyroid surgical position group compared to the supine position group (96.47% vs 85.37%; RR 1.13, 95% CI 1.03–1.24; *P* = 0.0145). No statistically significant difference was observed in median V1 amplitude between groups (thyroid surgical position group: 830 μV [630–1266.5] vs supine group: 825.5 μV [563.5–1421.25]; *P* = 0.8238).

**Table 2:** **Intraoperative nerve monitoring** and airway management outcomes

Variable	Thyroid surgical position (*n* = 85)	Supine position (*n* = 82)	Median difference [95% CI]/relative risk [95% CI]	*P*-value
V1 amplitude > 500 μV	82 (96.47%)	70 (85.37%)	1.13 [1.03–1.24]	0.0145
V1 amplitude, μV	830 (630–1266.5)	825.50 (563.50–1421.25)	4.5 [−191, 175.99]	0.8238
First attempt success	85 (100%)	82 (100%)	–	>0.999
Tracheal intubation time,s	54 (50–56)	55 (50–60)	−1 [−4.00, 1.5]	0.118
Cormack–Lehane grade				0.4505
Grade 1	65 (76.47%)	67 (81.71%)	0.94 [0.80–1.09]	
Grade 2	20 (23.53%)	15 (18.29%)	1.29 [0.72–2.30]	
IDS >5	3(3.53%)	8(9.76%)	0.36 [0.10–1.30]	0.2121
EMG tube depth, cm	22 (21–23)	22 (21–22)	1 [0, 1]	0.046

Data are presented as *n* (%), mean ± SD, or median (interquartile range). V1 amplitude, amplitude of vagus nerve stimulation before dissection; IDS, Intubation Difficulty Scale score; EMG tube depth, depth of the electromyography tube.

*Post hoc* multivariable regression analysis, adjusting for age, gender, and height, confirmed the robustness of our primary finding. The thyroid surgical position remained significantly associated with satisfactory EMG signals (adjusted RR = 1.12, 95% CI 1.02–1.24, *P* = 0.019). Age exerted a modest inverse effect (adjusted RR = 0.996, 95% CI 0.993–1.000, *P* = 0.039), while gender and height showed no significant effects (Table 3).

**Table 3:** Exploratory *post hoc* Poisson regression of factors associated with the primary outcome

Variable	*P*-value	Adjusted relative risk (95% CI)
Group (thyroid position vs supine position)	0.019	1.12 (1.02–1.24)
Age (per year)	0.039	0.996 (0.993–1.000)
Gender (male vs female)	0.835	0.98 (0.82–1.17)
Height (per cm)	0.162	0.99 (0.98–1.00)

All patients achieved successful tracheal intubation on the first attempt. The tracheal intubation time did not significantly differ between groups (54 s [50–56] vs 55 s [50–60]; *P* = 0.118). The thyroid surgical position group required a deeper EMG tube insertion compared to the supine position group (22 cm [21–23] vs 22 cm [21–22]; median differences 1, 95% CI 0–1; *P* = 0.046).

Regarding the Cormack–Lehane grades, no significant differences were observed between groups (*P* = 0.4505). Additionally, three patients (3.53%) in the thyroid surgical position group and eight patients (9.76%) in the supine position group had an IDS greater than 5, although this difference did not reach statistical significance (*P* = 0.2121; Table 2).

### Postoperative complications

Postoperative complications, including lip lesions, dental injury, postoperative nausea and vomiting, dizziness, and headache, were infrequent and did not differ significantly between groups (all *P* > 0.05; Table 4).

**Table 4:** Postoperative complications

Complication	Thyroid surgical position (*n* = 85)	Supine position (*n*=82)	Relative risk [95% CI]	*P*-value
Lip lesions, *n* (%)	1 (1.18)	2 (2.44)	0.48 [0.04–5.28]	0.6160
Dental injury, *n* (%)	0 (0.00)	0 (0.00)	/	>0.9999
PONV, *n* (%)	12 (14.12)	10 (12.20)	1.16 [0.53–2.54]	0.8203
Dizziness, *n* (%)	12 (14.12)	13 (15.85)	0.89 [0.44–1.82]	0.8298
Headache, *n* (%)	3 (3.53)	6 (7.32)	0.48 [0.12–1.99]	0.3231

Data are presented as *n* (%). PONV, postoperative nausea and vomiting.

### Clinician satisfaction

Clinician satisfaction scores were significantly higher in the thyroid surgical position group, with median ratings of 9 (9–10) compared to 5 (5–6) in the supine position group (median differences 4, 95% CI 3–4; *P* < 0.0001). Similarly, mask ventilation satisfaction was rated at 9 (8–9) in the thyroid surgical position group versus 8 (8–8) in the supine position group (median differences 1, 95% CI 0–1; *P* < 0.0001; Table 5).

**Table 5:** Satisfaction scores

Variable	Thyroid surgical position group (*n* = 85)	Supine position group (*n* = 82)	Median difference (95% CI)	*P*-value
Clinician satisfaction	9 (9–10)	5 (5–6)	4 [3, 4]	<0.0001
Mask ventilation satisfaction	9 (8–9)	8 (8–8)	1 [0, 1]	<0.0001

Data are presented as median (interquartile range).

## Discussion

This multicenter study confirms that performing tracheal intubation in the thyroid surgical position increases the probability of obtaining satisfactory IONM signals by 11 percentage points (96.47% vs 85.37%) compared with the conventional supine position. The absolute risk reduction translates into a number-needed-to-treat of ≈9, meaning that intubating nine patients in the thyroid surgical position yields one additional patient with a reliable V1 signal. The finding is clinically relevant because a ≥500 µV V1 amplitude is widely accepted as the minimum threshold for reliable RLN identification. Importantly, thyroid surgical position achieved this benefit without prolonging laryngoscopy time or increasing intubation difficulty, and it was associated with markedly higher clinician-rated satisfaction for both mask ventilation and patient positioning.

Accurate placement of the EMG tracheal tube is critical for successful IONM. Malposition can produce false-negative results or low-grade EMG signals, undermining surgical decision-making^[4]^. Previous reports show that 3.8–23% of patients experience signal-quality issues attributable to tube displacement^[9,10]^. Head-and-neck movement after intubation is a major culprit: fluoroscopic studies have documented up to 21 mm proximal and 33 mm distal migration when moving from neutral to maximal extension, i.e., a potential 60 mm total shift^[11–13]^. In most medical institutions, the standard protocol for thyroid surgery involves induction of anesthesia and endotracheal intubation with the patient in the supine position, followed by repositioning to the specific surgical position for thyroidectomy. Although the endotracheal tube is carefully secured intraoperatively to maintain the distance from the incisors unchanged, prior studies have confirmed that changes in head and neck position (e.g., during repositioning) can induce relative displacement of the tube within the tracheal lumen^[6,7,11]^. This displacement alters the contact location between the neuromonitoring catheter electrodes and the vocal cords, thereby reducing the quality of IONM signals.

To address this issue, the present study employs an improved approach that induction of anesthesia and endotracheal intubation in the fully extended thyroid surgery position. Through this method, we can effectively “pre-compensate” for movement-induced displacement, thereby maintaining stable electrode-glottis contact throughout the procedure. Notably, this approach does not increase the difficulty of intubation – evidenced by comparable intubation times (54 vs 55 seconds). There was also no significant difference in the distribution of Cormack–Lehane grades between the two groups. The absence of significant differences in median V1 amplitudes (830 vs 825.5 µV) indicates that the benefit is driven by fewer amplitudes dropping below the 500 µV cutoff, not by globally higher voltages. This confirms that stable electrode-tissue contact achieved through pre-positioned intubation is key to maintaining reliable IONM signals.

The thyroid surgical position group achieved a 96.47% rate of high-quality V1 signals, closely matching the values reported in earlier retrospective series^[14]^. By contrast, the supine group reached 85.37%, underscoring that precise tube placement in the thyroid surgical position markedly lowers the risk of electrode displacement during subsequent patient manipulation. These data reinforce the concept that positional tube migration is a principal cause of IONM signal degradation. Prior work has shown that even minor changes in patient posture, or shifts in thyroid and laryngeal anatomy, can alter the relationship between the EMG tube and the glottis^[15–17]^. Because of this instability, many early investigations recommended a second laryngoscopy, after head–neck extension, to reconfirm tube location^[18]^. Collectively, current and previous findings indicate that perioperative team have two options to secure optimal electrode contact: (1) perform a second laryngoscopy for readjustment or (2) intubate once in the fully prepared thyroid surgical position. The latter approach is clearly simpler, avoids additional airway instrumentation, and, as our data show, delivers equally reliable – and arguably superior – signal quality.

The *post hoc* analysis incidentally observed a minor age effect, with signal probability decreasing by 0.4% per year (adjusted RR = 0.996). While statistically significant, this translates to only 4% lower signals in a 60- versus 40-year-old patient – unlikely to impact clinical decision-making. The effect may reflect age-related laryngeal tissue changes, though confirmation requires dedicated studies.

Mask-ventilation satisfaction was higher in the thyroid surgical position group, likely because the posture closely replicates the classic “sniffing” alignment, straightening the oral–pharyngeal–laryngeal axes and improving airway patency. Despite the full extension, video-laryngoscopic glottic exposure, IDS difficulty scores, and intubation times were comparable between groups, confirming that thyroid surgical position does not make laryngoscopy more challenging. The substantially higher staff-satisfaction scores in the thyroid surgical position group probably reflect the logistics advantage of positioning patients while they are still awake and cooperative rather than after induction-of-anesthesia muscle relaxation – an issue that becomes especially relevant in heavier patients.

EMG amplitude is exquisitely sensitive to the degree of neuromuscular blockade, whereas inhalational and intravenous anesthetics have negligible impact^[15,19]^. We therefore standardized induction with a single ED₉₅ dose (0.3 mg·kg^−1^) of rocuronium – shown to preserve satisfactory EMG signals within 20 min and withheld further doses^[20–24]^. This protocol isolates patient position as the principal determinant of signal quality in the present analysis. Future studies might explore whether routine sugammadex reversal can further buffer against low-amplitude outliers.

This study has several limitations. First, restricted BMI (18–28 kg·m^−2^) limits generalizability to obese patients, in whom redundant soft tissue may alter both laryngoscopy dynamics and tracheal elasticity. Second, this study did not measure the neck length of patients, a variable that may influence the degree of endotracheal tube displacement after intubation. Future studies should incorporate standardized assessments of cervical anatomical parameters (e.g., neck circumference, cervical spine mobility, and neck length) to optimize positional selection criteria, taking into account individual variability. Third, while improved signal reliability reduces false-negative monitoring risk, future studies should correlate this with RLN injury rates. Nevertheless, maintaining amplitude >500 µV is critical for valid IONM according to international guidelines. Fourth, the absence of quantitative TOF monitoring represents a study constraint. However, our standardized rocuronium dosing (0.3 mg·kg^−1^) aligns with evidence demonstrating preserved laryngeal EMG signals at this dose^[20–24]^. Finally, all intubations were performed by experienced anesthetists using a single video-laryngoscopic system; results may differ with less-experienced operators or alternative devices.

## Conclusion

Intubating in the thyroid surgical position significantly enhances RLN monitoring signals compared with the supine position. This approach also improves mask ventilation effectiveness while maintaining intubation simplicity, offering a straightforward, beneficial technique for thyroid surgery.

## Data Availability

The datasets used and/or analyzed during the current study are available from the corresponding author upon reasonable request.

## Research registration unique identifying number (UIN)

The study has been registered in the Clinical Trial Registration Center of China (Registration No.: ChiCTR2400091542).

## References

[1] Dou Z Shi Y Jia J. Global burden of disease study analysis of thyroid cancer burden across 204 countries and territories from 1990 to 2019. Front Oncol 2024;14:1412243. 38873252 10.3389/fonc.2024.1412243 PMC11175622

[2] Jeannon J-P Orabi AA Bruch GA Abdalsalam HA Simo R. Diagnosis of recurrent laryngeal nerve palsy after thyroidectomy: a systematic review. Int J Clin Pract 2009;63:624–29. 19335706 10.1111/j.1742-1241.2008.01875.x

[3] Sun H Tian W, College of Surgeons CM. Chinese guidelines on intraoperative neuromonitoring in thyroid and parathyroid surgery (2023 edition). Gland Surg 2023;12:1031. 37701297 10.21037/gs-23-284 PMC10493630

[4] Barber SR Liddy W Kyriazidis N. Changes in electromyographic amplitudes but not latencies occur with endotracheal tube malpositioning during intraoperative monitoring for thyroid surgery: implications for guidelines. Laryngoscope 2017;127:2182–88. 27861939 10.1002/lary.26392

[5] Lu I-C Lin I-H Wu C-W. Preoperative, intraoperative and postoperative anesthetic prospective for thyroid surgery: what’s new. Gland Surg 2017;6:469–75. 29142836 10.21037/gs.2017.05.02 PMC5676182

[6] Tailleur R Bathory I Dolci M Frascarolo P Kern C Schoettker P. Endotracheal tube displacement during head and neck movements. Observational clinical trial. J Clin Anesth 2016;32:54–58. 27290945 10.1016/j.jclinane.2015.12.043

[7] Kang DH Kim SH You HE Kim WM. Is endotracheal tube displacement during head and neck extension due to ascending movement or tracheal lengthening? An observational ultrasonographic study. J Clin Monit Comput 2023;37:139–45. 35616797 10.1007/s10877-022-00870-w

[8] TITAN Group, Agha RA Mathew G Rashid R. Transparency in the reporting of Artificial Intelligence– the TITAN guideline. Prem J Sci 2025;10:100082.

[9] Ic L Ks C Cj T. Optimal depth of NIM EMG endotracheal tube for intraoperative neuromonitoring of the recurrent laryngeal nerve during thyroidectomy. World J Surg 2008;32:1935–1939. 18392652 10.1007/s00268-008-9549-1

[10] Beldi G Kinsbergen T Schlumpf R. Evaluation of intraoperative recurrent nerve monitoring in thyroid surgery. World J Surg 2004;28:589–91. 15366750 10.1007/s00268-004-7226-6

[11] Yap SJ Morris RW Pybus DA. Alterations in endotracheal tube position during general anaesthesia. Anaesth Intensive Care 1994;22:586–88. 7818064 10.1177/0310057X9402200515

[12] Kim J-H Hong D-M Oh A-Y Han S-H. Tracheal shortening during laparoscopic gynecologic surgery. Acta Anaesthesiol Scand 2007;51:235–38. 17181537 10.1111/j.1399-6576.2006.01208.x

[13] Jin-Hee K Ro Y-J Seong-Won M. Elongation of the trachea during neck extension in children: implications of the safety of endotracheal tubes. Anesth Analg 2005;101:974–77. 16192505 10.1213/01.ane.0000169330.92707.1e

[14] Huang J-M Hsu C-D Wu S-H. Optimization of electromyographic endotracheal tube electrode position by UEScope for monitored thyroidectomy. Laryngoscope Invest Otolaryngol 2021;6:1214–19. 10.1002/lio2.635 PMC8513421 34667867

[15] Randolph GW Dralle H Abdullah H. Electrophysiologic recurrent laryngeal nerve monitoring during thyroid and parathyroid surgery: international standards guideline statement. Laryngoscope 2011;121:S1–16. 21181860 10.1002/lary.21119

[16] Liddy W Barber SR Cinquepalmi M. The electrophysiology of thyroid surgery: electrophysiologic and muscular responses with stimulation of the vagus nerve, recurrent laryngeal nerve, and external branch of the superior laryngeal nerve. Laryngoscope 2017;127:764–71. 27374859 10.1002/lary.26147

[17] Tsai C-J Tseng K-Y Wang F-Y. Electromyographic endotracheal tube placement during thyroid surgery in neuromonitoring of recurrent laryngeal nerve. Kaohsiung J Med Sci 2011;27:96–101. 21421197 10.1016/j.kjms.2010.08.002 PMC11916461

[18] Kanotra SP Kuriloff DB Lesser J Rest-Flarer F. GlideScope-assisted nerve integrity monitoring tube placement for intra-operative recurrent laryngeal nerve monitoring. J Laryngol Otol 2012;126:1271–73. 23098106 10.1017/S0022215112002460

[19] Marusch F Hussock J Haring G Hachenberg T Gastinger I. Influence of muscle relaxation on neuromonitoring of the recurrent laryngeal nerve during thyroid surgery. Br J Anaesth 2005;94:596–600. 15734779 10.1093/bja/aei110

[20] Lu I-C Tsai C-J Wu C-W. A comparative study between 1 and 2 effective doses of rocuronium for intraoperative neuromonitoring during thyroid surgery. Surgery 2011;149:543–48. 21236452 10.1016/j.surg.2010.11.006

[21] Lu I-C Wu C-W Chang P-Y. Reversal of rocuronium-induced neuromuscular blockade by sugammadex allows for optimization of neural monitoring of the recurrent laryngeal nerve. Laryngoscope 2016;126:1014–19. 26748952 10.1002/lary.25577

[22] Han Y Liang F Chen P. Dosage effect of rocuronium on intraoperative neuromonitoring in patients undergoing thyroid surgery. Cell Biochem Biophys 2015;71:143–46. 25120022 10.1007/s12013-014-0176-1

[23] Garofalo E Bruni A Scalzi G. Low-dose of rocuronium during thyroid surgery: effects on intraoperative nerve-monitoring and intubation. J Surg Res 2021;265:131–38. 33940235 10.1016/j.jss.2021.03.041

[24] Ic L Sh W Py C. Precision neuromuscular block management for neural monitoring during thyroid surgery. J Invest Surg 2021;34:1389–1396. 32791867 10.1080/08941939.2020.1805055

